# Tree seed traits’ response to monsoon climate and altitude in Indian subcontinent with particular reference to the Himalayas

**DOI:** 10.1002/ece3.3181

**Published:** 2017-08-11

**Authors:** Surendra P. Singh, Shyam S. Phartyal, Sergey Rosbakh

**Affiliations:** ^1^ Central Himalayan Environment Association (CHEA) 06 Waldorf Compound Nainital Uttarakhand India; ^2^ Department of Forestry and Natural Resource HNB Garhwal University Srinagar‐Garhwal Uttarakhand India; ^3^ Chair of Ecology and Conservation Biology University of Regensburg Regensburg Germany

**Keywords:** altitudinal gradient, climate change, ecophysiology, Himalayas, monsoon, seed desiccation, seed dispersal, seed germination ecology, tree

## Abstract

Seed traits are related to several ecological attributes of a plant species, including its distribution. While the storage physiology of desiccation‐sensitive seeds has drawn considerable attention, their ecology has remained sidelined, particularly how the strong seasonality of precipitation in monsoonal climate affects their temporal and spatial distribution. We compiled data on seed mass, seed desiccation behavior, seed shedding, and germination periodicity in relation to monsoon and altitude for 198 native tree species of Indian Himalayas and adjoining plains to find out (1) the adaptive significance of seed mass and seed desiccation behavior in relation to monsoon and (2) the pattern of change in seed mass in relation to altitude, habitat moisture, and succession. The tree species fall into three categories with respect to seed shedding and germination periodicities: (1) species in which both seed shedding and germination are synchronized with monsoon, referred to as monsoon‐synchronized (MS, 46 species); (2) species in which seed germination is synchronized with monsoon, but seeds are shed several months before monsoon, referred to as partially monsoon‐synchronized (PMS, 112 species); and (3) species in which both shedding and germination occur outside of monsoon months, referred to as monsoon‐desynchronized (MD, 39 species). The seed mass of MS species (1,718 mg/seed) was greater than that of PMS (627 mg/seed) and MD (1,144 mg/seed). Of the 40 species with desiccation‐sensitive seeds, 45% belong to the MS category, almost similar (approx. 47%) to woody plants with desiccation‐sensitive seeds in evergreen rain forests. Seed mass differed significantly as per seed desiccation behavior and successional stage. No relationship of seed mass was found with altitude alone and on the basis of seed desiccation behavior. However, seed mass trend along the altitude differed among monsoon synchronization strategies. Based on our findings, we conclude that in the predicted climate change (warming and uncertain precipitation pattern) scenario, a delay or prolonged break‐spell of monsoon may adversely affect the regeneration ecology of desiccation‐sensitive seed‐bearing species dominant over large forest areas of monsoonal climate.

## 1 INTRODUCTION

Seed mass and seed desiccation sensitivity are key life‐history traits of high ecological relevance (Leishman, Wright, Moles, & Westoby, 2000). For example, seed mass (the mass of the embryo, endosperm, and seed coat; Usher, 1966) influences seed dispersal and seedling survival rates, particularly under low‐light conditions, and other species responses related to regeneration (Grime & Jeffrey, 1965; Murali, 1997; Walker & Reich, 2000; Khurana & Singh, 2001; Moles et al., 2007). Seed mass is also correlated with many central life‐history traits including plant size, time required to achieve first reproduction, and life span (Moles, Falster, Leishman, & Westoby, 2004), and with the number of seeds a plant can make for a given amount of canopy in a year (Aarssen & Jordan, 2001; Henery & Westoby, 2001). Furthermore, seed mass is related to the sensitivity of seeds to desiccation, the water content of seeds, and time that seeds take to germinate, and seedling survival (Doussi & Thanos, 2002; Hong & Ellis, 1998; Leishman et al., 2000; Pritchard et al., 2004). With regard to desiccation sensitivity, seeds are divisible into two broad categories: (1) desiccation‐sensitive seeds which are dispersed with high moisture content, typically above 40% on fresh‐weight basis, and cannot survive drying to water content even as high as 20%–30% on fresh‐weight basis; and (2) desiccation‐tolerant seeds, which can tolerate drying to low (below 7%) water contents with hardly any effect on viability (Pritchard, 2004; Roberts, 1973). Due to difficulty in storage, the desiccation‐sensitive seeds are called “recalcitrant,” while the desiccation‐tolerant seeds are referred to as “orthodox.” Between these two, there is an “intermediate” category in which seeds tolerate desiccation more than recalcitrant seeds although their tolerance is much more limited than that of orthodox seeds (Ellis, Hong, & Roberts, 1990). As per Tweddle, Turner, and Dickie (2002) and Tweddle, Dickie, Baskin, and Baskin (2003), of the approximately 8,000 seed‐bearing species that have been investigated, only about 640 species have desiccation‐sensitive seeds, and the remaining 7,360 (about 92%) have desiccation‐tolerant seeds. In previous studies, seed desiccation sensitivity was found to be correlated with several ecological traits such as seed dispersal in the wet season, nondormancy at seed stage, rapid germination after shedding to avoid seed perdition, and the ability to persist as a seedling banks (Daws, Garwood, & Pritchard, 2005; Garwood, 1989; Pritchard et al., 2004; Tweddle et al., 2003; Vázquez‐Yanes & Orozco‐Segovia, 1993), get the advantage of large‐seededness by young seedlings when still attached to the parent seed to renew growth after herbivore damage (Forget, 1992), and influence the success of recruitment and hence vegetation composition (Joet, Ourcival, & Dussert, 2013). A detailed knowledge of the ecology of seed desiccation sensitivity will help to understand the role that this trait plays in regeneration ecology (Dickie & Pritchard, 2002).

Although physiological and biochemical aspects of desiccation‐sensitive seeds have been investigated extensively (Pammenter & Berjak, 2000), particularly because they cannot be stored for a long period, their ecological aspects have been little investigated. Generally, desiccation‐sensitive or desiccation‐tolerant seeds could explain differences in species distribution patterns, because they may be affected by climate (Poschlod et al., 2013). Specifically, species with desiccation‐sensitive seeds are quite common in the forests of moist climate, particularly rain forest and their percentage declines from tropical to the temperate regions and from wet to arid areas (Tweddle et al., 2003). They also argue with cautions that in a highly seasonal environment, species with desiccation‐sensitive seed are less common and they tend to disperse their seeds during wet period of the year. Desiccation‐sensitive seeds do not undergo maturation drying and the water content does not diminish at all during seed development (Berjak, Farrant, Mycock, & Pammenter, 1990). Seeds germinate shortly after dispersal with the available water upon shedding until the water becomes a limiting factor (Farrant, Pammenter, & Berjak, 1988). Furthermore, the desiccation‐sensitive species had large seeds with “thin” seed coats (Daws, Garwood & Pritchard, 2006). These characteristics make desiccation‐sensitive seeds highly susceptible to desiccation damage if field conditions are not conducive.

From a taxonomical point of view, the desiccation sensitivity is associated with certain families like *Dipterocarpaceae* and *Fagaceae* (Dickie & Pritchard, 2002). In the Himalayas and adjoining plains of the Indian subcontinent, some of the desiccation‐sensitive species cover large areas, *Shorea robusta* (*Dipterocarpaceae*) in the plains and several oaks (*Quercus* spp., *Fagaceae*) in the Himalayas being the typical examples (Singh & Singh, 1992). However, given that the monsoon climate is characterized by about three to four wet months, when about 75% of annual precipitation occurs and frequent spells of drought during the rest of the year, the ecological domination (in terms of geographical area of coverage, tree density, and tree basal area in stands in which they occur) of desiccation‐sensitive seed‐bearing tree species is rather unexpected (Joet et al., 2013) and warrants investigations.

Because seed mass influences seed germination and seedling growth, several studies have been carried out to determine the patterns of seed mass along latitudinal and altitudinal gradients. While most studies have observed a decrease in seed mass with increasing latitude (Moles et al., 2007), the picture along an altitudinal gradient is inconsistent. With increasing altitude seed mass is reported to increase (Ayana & Bekele, 2000; Pluess, Schütz, & Stöcklin, 2005), as well as decrease (Wang et al., 2014) or show no change (Holm, 1994). Furthermore, seed mass varies in relation to ecological succession, with seeds being larger in late succession species than early succession species (Moles et al., 2005; Salisbury, 1974). Seed mass also shows a positive correlation with other ecological factors such as temperature, precipitation, net primary productivity (NPP), leaf area index (LAI), seed dispersal syndrome, and plant growth form (Moles et al., 2005, 2014).

Trees in monsoon climate of the Indian subcontinent vary in the periodicity of seed shedding and seed germination in relation to monsoon months, typically from June to September (Singh & Singh, 1992), thus offer an opportunity to examine how the variation in the period when seeds remain lying on the ground and desiccate, seed mass and seed desiccation sensitivity are interrelated. This study is focused on forest trees of northern regions of the Indian subcontinent, particularly Himalayas and adjoining plains and attempts to shed more light on (1) the adaptation of seed mass and seed desiccation behavior to the monsoon pattern of precipitation and (2) the pattern of change in seed mass in desiccation‐sensitive/tolerant species in relation to the altitude, habitat moisture, and succession. Firstly, we hypothesize that the percentage of species with desiccation‐sensitive seeds will be higher in the species group in which both seed shedding and germination are linked with monsoon than in others in which the monsoon linkage is weak. In a highly favorable (warm and wet) monsoon season, species will maximize seedling recruitment and growth by shedding seeds during the rainy season or close to it and germinating quickly. As desiccation‐sensitive seeds are shed in a hydrated and metabolically active condition (Tweddle et al., 2003), they can maximize taking the advantage of the favorable monsoon condition for root growth before the onset of winter. Secondly, we hypothesize that the average seed mass will be greater in desiccation‐sensitive species, hence in those species in which monsoon synchronization is strong. Both large seeds and desiccation sensitivity are favored by similar conditions (Farnsworth, 2000; Grubb, Newbery, Prins, & Brown, 1998). Thirdly, we hypothesize that the upper limit of the altitudinal range of species with desiccation‐tolerant seeds will be higher than of the species with desiccation‐sensitive seeds because species with desiccation‐tolerant seeds are more adapted to stressful conditions (Berjak, 2006). In the Himalayas, generally, precipitation increases up to 1,000 m and then declines at higher altitudes (Friend & Woodward, 1990). As plant successional stage associated closely with seed mass and seed desiccation behavior (Dickie & Pritchard, 2002; Vázquez‐Yanes, Orozco‐Segovia, Sánchez‐Coronado, Rojas‐Aréchiga, & Batis, 2000 **)**, we finally hypothesize that the average seed mass and frequency of species with desiccation‐sensitive seeds will be higher among late‐successional tree species.

## 2 MATERIALS AND METHODS

**Figure 1:**
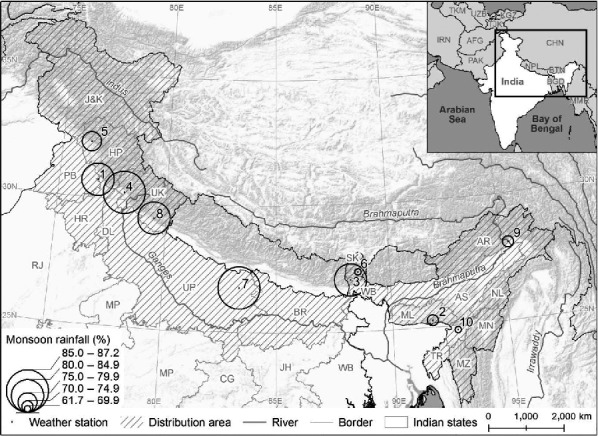
The Indian Himalayas and adjoining plains illustrating species distribution areas, locations of weather stations and intensity of monsoon rainfall (mm) as a percentage of total annual rainfall

The study region includes the Himalayas, their foothills, and the adjoining plains mostly with the monsoon pattern of rainfall. To represent a typical monsoonal rainfall pattern, monthly rainfall for 25–100 years period (based upon 1901–2000 data; Indian Meteorological Department, 2015) of several weather locations covering entire species distribution range is presented in Fig. 1. Three to four months of monsoon (typically from mid‐June to the end of September when total precipitation received is about 75% of the annual rainfall) is followed by spells of the dry period during winter and early summer or pre‐monsoon season (March to mid‐June), interrupted occasionally by rain showers. The amount of precipitation varies widely, some areas in the north of main Himalayan ranges receive less than 500 mm precipitation annually, while on slopes south of the main Himalayan ranges the annual precipitation typically ranges from 1,500 to 3,000 m (Immerzeel, Petersen, Ragettli, & Pellicciotti, 2014). As for the recent climatic changes, Himalayan regions are warming at rates 3–5 times higher than global average rate and the extent of temperature rise increases with altitude (Shrestha, Gautam, & Bawa, 2012). In addition, monsoon has weakened over last two to three decades in much of the Himalayas (Bhuiyan, Flügel, & Singh, 2009; Yao et al., 2012).

Generally, forests occur up to 4,000 m altitude, and consist of diverse tree species: *Shorea robusta,* a dipterocarp in foothills, subtropical pines, like *Pinus roxburghii* between 1,000 and 2,000 m, and several evergreen oaks (*Quercus* spp.) from 1,000 to >3,000 m altitude. Conifers, particularly species of *Abies, Cedrus, Cupressus, Picea*, and *Pinus* (*Pinus wallichiana* and *Pinus gerardiana)* dominate higher ranges >2,000 m, including areas beyond the direct thrust of monsoon. Treelines between 3,000 and 4,000 m generally consist of *Betula utilis*,* Abies spectabilis*, and *Rhododendron campanulatum* (a krummholz species).

### 2.1 Data collection

We collected information about several ecological traits, viz. altitudinal range, habitat moisture type, a response of mature seeds to desiccation sensitivity/tolerance, seed mass, seed shedding and germination timing, and succession stage, for 198 tree species representing 51 families and 116 genera (Appendix S1). Information on the timing of seed shedding and seedling emergence (hereafter referred to as seed germination), habitat moisture type, such as dry and moist, and succession stage was largely gathered from classical work of Troup (1921) on “Silviculture of Indian Trees,” from Champion and Seth (1968), Ralhan, Khanna, Singh, and Singh (1985), Rao (1984), Singh and Singh (1987, 1992), and Thapliyal and Phartyal (2005). The timings of seed shedding and seed germination were shown as a range of months. The main sources for data on seed mass and seed desiccation behavior were Khullar, Thapliyal, Beniwal, Vakshasya, and Sharma (1991), Rao (1984), Royal Botanical Garden Kew Seed Information Database (SID, 2015), Troup (1921), Thapliyal, Thapliyal, Bahar, Naithani, and Bist (2003), and several other published Indian Ph.D. theses and research papers on seed biology of Indian trees. To make data easily comparable, species with “intermediate” seed storage physiology (only four species in our database) were grouped together with species having “orthodox” seeds, as both categories of seeds essentially tolerate a different degree of desiccation. In Kew SID, species are also categorized as “probable orthodox” considering their ability to withstand desiccation. In the present arrangement, all species with “probable orthodox” seeds along with “orthodox” and “intermediate” categories were grouped together as desiccation‐tolerant seeds. All species with seed storage physiology of “recalcitrant” and/or “recalcitrant? {a species is likely (greater than evens chance), rather than certain, to display a recalcitrant storage behavior as per Kew SID judgment}” were grouped as desiccation‐sensitive seeds for analysis. Species with uncertain seed storage physiology were not considered for analysis in this study.

With regard to succession, species were placed into two broad categories: (1) early succession and (2) late succession. Information about the timing of seed shedding and seed germination that we collected from various sources were arranged and categorized in reference to the timing of monsoon precipitation pattern. For seed shedding and germination each species was assigned a range of months, primarily from Ralhan et al. (1985), Singh and Singh (1992) and Troup (1921). Generally, seed shedding and germination in a species either occurred during monsoon or outside of monsoon months, so assigning a species to the categories in relation to monsoon was not difficult. In some species, seeds that failed to germinate during the monsoon season that followed seed shedding germinated only during the second‐ or third‐, or even fourth‐year monsoon season. Although no separate category was created for them, information on the existence of such species was collected from Troup (1921). The timing of seed shedding of a species could vary from one place to another, particularly when altitude differs. In such a situation, information from the midpoint of altitudinal range was taken. Species predominantly occurring in moist and dry forest types as per the classification of Champion and Seth (1968) were divided into moist and dry habitat types, respectively. Those occurring frequently in both moist and dry types were classified as intermediate habitat type.

In total, we could collect data for 198 commonly occurring tree species (representing approximately one‐third of tree species distributed in our study regions) on altitudinal range and seed mass, 197 species on the timings of seed shedding and seed germination, 190 species on seed desiccation sensitivity/tolerance and for 166 and 111 species on habitat moisture types and succession stage, respectively.

### 2.2 Species categorization in relation to monsoon synchronization strategy and seed traits

As for the timing of seed shedding and seed germination in response to monsoon period, species were divided into the following three categories: (1) species in which both seed shedding and seed germination occurred broadly at the same time during monsoon months, referred to as monsoon‐synchronized (MS) species; (2) species in which seeds were shed several months before the onset of monsoon (generally during autumn and winter months), but seed germination occurred during monsoon months, referred to as partially monsoon‐synchronized (PMS) species; and (3) species in which both seed shedding and seed germination occurred outside of monsoon months, referred to as monsoon‐desynchronized (MD) species.

### 2.3 Data analysis

As related species are likely to share similar attributes (Harvey & Pagel, 1991), prior to estimating the seed mass variation among the different species groups, we calculated an estimated value of *K*, a Brownian motion‐based metric of the strength of phylogenetic signal (Blomberg, Garland, & Ives, 2003) using the *phylosignal* function in the “picante” library (Kembel et al., 2010). *K *= 1 indicates that closely related species have trait values that are similar to those expected given Brownian motion; *K* < 1 indicates that closely related species have trait values that are less similar than expected given a Brownian model of evolution. In order to estimate the phylogenetic relationships among the study species, which are required for the *K*‐statistics, we assembled a phylogenetic tree with the help of Phylomatic v3 (Webb & Donoghue, 2005), available at http://phylodiversity.net/phylomatic/, based on the maximally resolved tree (Zanne et al., 2014).

As the phylogenetic signal was not detected in any of the groups tested, we used generalized least squares (GLS) regression models to estimate the seed mass variation among the different species groups in relation to monsoon synchronization strategy, habitat moisture, succession, seed desiccation behavior, and seed mass variation along altitudinal gradient. This type of regression was chosen due to its ability to account for heteroscedasticity that was a problem in the majority of the data sets used for the analysis. In all regressions calculated, we used seed mass as a responsible variable and grouping factor of interest (e.g., “dry,” “intermediate,” “moist” for habitat as per availability of moisture) as an explanatory variable. As for the relationship between seed mass, seed desiccation behavior, and monsoon synchronization strategy along the altitudinal gradient, we set up three GLS regressions. As explanatory variables for seed mass, we used altitude in the first model, seed desiccation behavior and monsoon synchronization strategy in the second and third models, respectively. In the latter two cases, the explanatory variables were included in the model as interaction with the altitude. Log(*x* + 1) transformation was applied to the data on seed mass, in order to obtain more normal residuals. All statistical calculations were performed in R software version 3.2.0 (R Core Development Team, 2016).

## 3 RESULTS

Of the 198 species for which information could be collected for the timing of seed shedding and seed germination, 46 belonged to MS, 112 to PMS, and 39 to MD (Table 1). The MS category included many broad‐leaved species of moist habitats, such as the species of *Cinnamomum, Litsea*,* Machilus, Quercus*, and *Shorea*. In much of the altitudinal range that supports forest vegetation in the Himalayas, one or more species of the MS category dominate, for example, *Quercus floribunda, Q. semecarpifolia* and *Shorea robusta*. Seeds in about 42% of these species had a short viability, and their seeds were sensitive to desiccation. The PMS category included the majority of species (*Bauhinia* spp.*, Cassia fistula, Phyllanthus emblica*, and *Pinus roxburghii)* with desiccation‐tolerant seeds, only about 18% of the species had desiccation‐sensitive seeds. The MD category included several high‐altitude (above 2,000 m) Himalayan species (*Abies* spp., *Acer* spp., *Aesculus indica, Cedrus deodara, Fraxinus* spp., *Picea* spp., and *Taxus baccata*) where snowmelt supplies water for germination. About 8% of these species belong to desiccation‐sensitive seeds (Table 1). Of the 188 species for which both monsoon synchronization and seed desiccation information were available, desiccation‐sensitive seeds found only in 40 species, and 45% of them belong to the MS, 47% to the PMS, and 8% to the MD category.

**Table 1:** Number and percentage of tree species as per monsoon synchronization categories (MS, monsoon‐synchronized; PMS, partially monsoon‐synchronized; MD, monsoon‐desynchronized) and species with desiccation‐sensitive (DS) and desiccation‐tolerant (DT) seeds in three categories

Monsoon synchronization category	Total species (no. and %)	Species as per seed desiccation behavior		
		DS seeds (no. and %)	DT seeds (no. and %)	Total
MS	46 (23.3)	18 (41.9)	25 (58.1)	43
PMS	112 (56.8)	19 (17.6)	89 (82.4)	108
MD	39 (19.8)	03 (8.1)	34 (91.9)	37
Total	197	40 (21.3)	148 (78.7)	188

The seed mass values of the studied species ranged from 0.02 mg/seed (*Duabanga grandiflora*, a deciduous fast growing tree of Eastern Himalayas) to 23,994 mg (*Aesculus indica*, a deciduous and dominant tree of temperate Himalayas, growing up to about 3,000 m alongside oaks, maples, birches and laurels), displaying a median of 107.2 mg. The 5th and 95th percentiles of the overall distribution of our data showed that the seed mass values of the species included in the analysis ranged from 0.50 to 4,841 mg/seed.

### 3.1 Phylogenetic signal in the seed mass

A phylogenetic signal was not detected in any of the species groups tested, indicating a lack of association of the seed mass with phylogeny (Table 2).

**Table 2:** Phylogenetic conservatism in seed mass and species group according to Bloomberg's *K*‐statistics (for details see Section 2.3). *K *= 1 indicates that closely related species have trait values that are similar to those expected given Brownian motion. *K *< 1 indicates that closely related species have trait values that are less similar than expected given a Brownian model of evolution. *p*, significance value

Species group	*K*‐statistic	*p*
All species	0.05	.63
Monsoon synchronization strategy		
MS	0.2	.7
PMS	0.02	.89
MD	0.23	.36
Habitat moisture		
Dry	0.15	.52
Intermediate	0.38	.44
Moist	0.08	.73
Succession stage		
Early succession	0.24	.21
Late succession	0.08	.58
Seed desiccation behavior		
Desiccation‐sensitive	0.05	.34
Desiccation‐tolerant	0.16	.17

### 3.2 Seed mass and monsoon synchronization strategy

**Figure 2:**
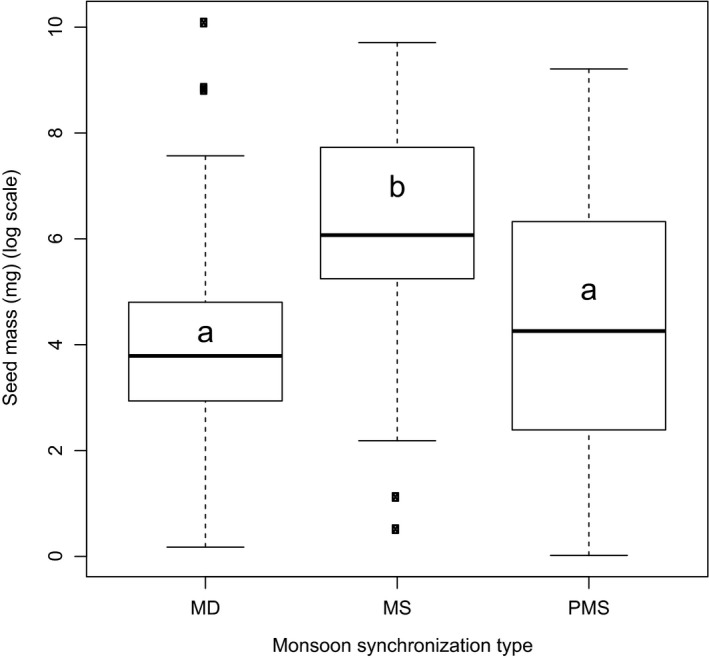
Box plots illustrating median, quartiles, and outliers (o) of mean seed mass of tree species as per their monsoon synchronization strategy. MS, monsoon‐synchronized (*n *= 46); PMS, partially monsoon‐synchronized (*n* = 112); MD, monsoon‐desynchronized (*n* = 39). Letters represent subsets with significant (*p* < .001) differences

The GLS regression revealed that seed mass of the MS category (averages seed mass 1,718 ± 570 mg/seed; *n* = 46) was significantly larger than that of MD (1,144 ± 422 mg/seed; *n* = 39) and PMS (627 ± 487 mg/seed; *n* = 112) species (Fig. 2).

### 3.3 Seed mass variation along altitudinal gradient

**Figure 3:**
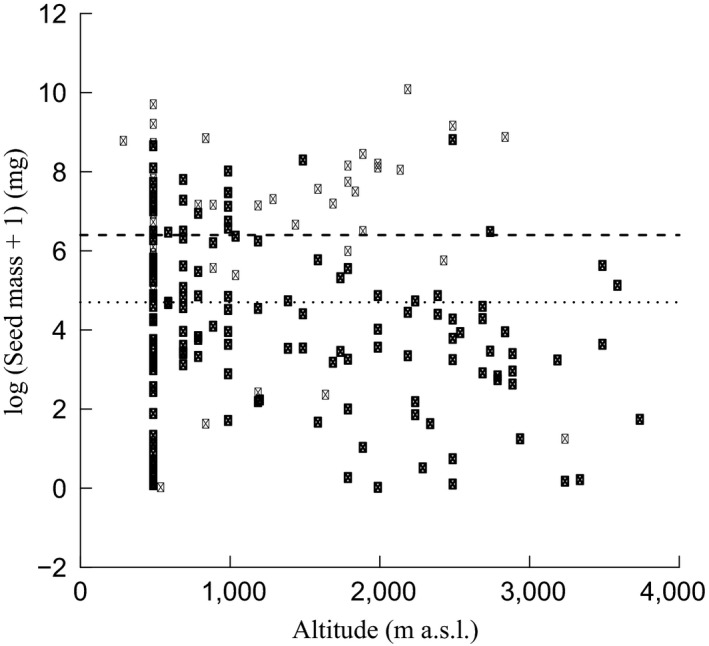
Regressions of log‐transformed seed mass to altitude with regard to the seed desiccation behavior (○: desiccation‐tolerant, *n* = 149; ●: desiccation‐sensitive, *n* = 41). For each group, the regression line is given (dotted line: desiccation‐tolerant; dashed line: desiccation‐sensitive)

**Figure 4:**
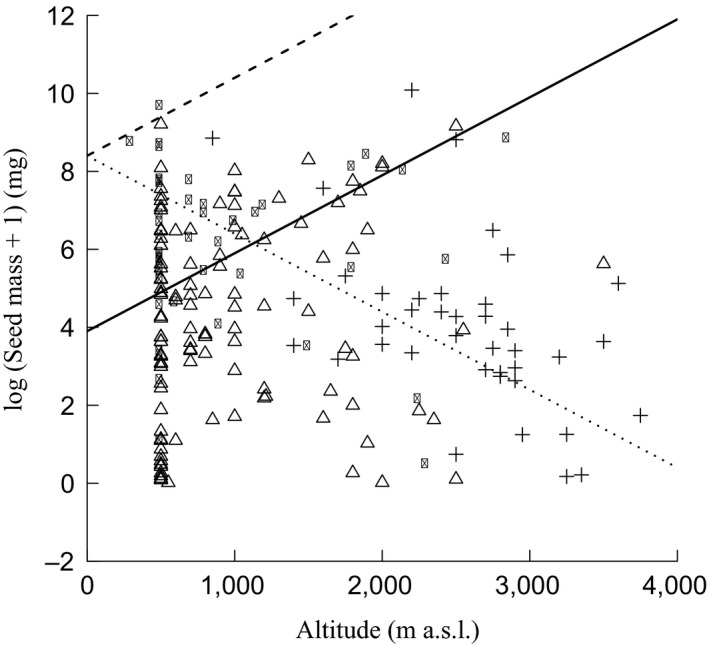
Regressions of log‐transformed seed mass to altitude with regard to the monsoon synchronization category (Δ: monsoon‐synchronized [MS]; ●: partially monsoon‐synchronized [PMS]; and +: monsoon‐desynchronized [MD]). For each category, the regression line is given (dashed line: MS; solid line: PMS; and dotted line: MD)

The first GLS regression revealed that seed mass did not vary along the altitudinal gradient (intercept = 5.1 ± 0.3 (*p* < .001); slope = −0.0004 ± 0.0002 (*p* = .06)). The results of the second GLS regression (Table 3) confirmed that seed mass of desiccation‐tolerant species was significantly lower than their desiccation‐sensitive counterparts. Effect of altitude on seed mass of species with different desiccation behavior was not detected. As parameter estimations for intercepts (desiccation‐tolerant) were significant (Table 3), two regression models were set up: one for desiccation‐tolerant and another one for desiccation‐sensitive seeds (Fig. 3). The third GLS regression revealed that seed mass varied significantly along altitudinal gradient as per monsoon synchronization strategy (Table 4), thus, three regression models were set up; one each for MS, PMS, and MD (Fig. 4). Seed mass was found to be positively correlated with altitude for the MS and PMS categories, whereas seed mass of MD species decreased when moving along an altitudinal gradient. When we compared two or more species of a genus that occupied different altitudes in the Himalayas, we observed that the seed mass was generally higher in species of higher altitude than those of lower altitude (Table 5).

**Table 3:** Estimated parameters for intercepts (*B*) and slopes (*m*) of the species with seed desiccation behavior (output of the second linear model) for the seed mass–altitude relation. Based on significance for intercepts (desiccation‐sensitive seeds), new slopes (*m*′) and intercepts (*B*′) were derived to draw regression lines of the species with different desiccation behavior in Fig. 3. Significant *p‐*values are bold entries. *SE*, standard error

Seed desiccation behavior	*B*	*SE*	*p*‐Value	*m*	*SE*	*p*‐Value	*B*′	*m*′
Desiccation‐sensitive seeds	6.4	0.7	**<.001**	−0.0002	0.0004	.68	6.4	0.0
Desiccation‐tolerant seeds	4.7	0.7	**.016**	−0.0007	0.0005	.16	4.7	0.0

**Table 4:** Estimated parameters for intercepts (*B*) and slopes (*m*) of the species with different monsoon synchronization strategy (output of the third linear model) for the seed mass–altitude relation. Based on significance for intercepts and slopes (in both cases MD and PMS), new slopes (*m*′) and intercepts (*B*′) were derived to draw regression lines of the species with different monsoon synchronization strategy in Fig. 4. Significant *p*‐values are bold entries. *SE*, standard error

Monsoon synchronization category	*B*	*SE*	*p*‐Value	*m*	*SE*	*p*‐Value	*B*′	*m*′
MS	6.2	1.6	.17	0.002	0.008	**.047**	8.4	0.002
PMS	3.9	1.5	**.04**	0.002	0.007	**.002**	3.9	0.002
MD	8.4	1.5	**<.001**	−0.002	0.0006	**.003**	8.4	−0.002

**Table 5:** A comparison of seed mass between the pairs of related species in the Himalayas (in each species pair, one species occurred at low (L) and the other at high (H) altitude, in the case of pine and oak, there are two species in each altitudinal category)

Species pair	Altitude (m) (midpoint of the range)	Mean seed mass (mg/seed)
*Abies pindrow*	2,900 (L)	18.3
*Abies spectabilis*	3,500 (H)	37.0
*Acer oblongum*	1,400 (L)	33.3
*Acer caesium*	2,400 (H)	80.0
*Betula alnoides*	2,500 (L)	0.11
*Betula utilis*	3,250 (H)	0.19
*Pinus kesiya*	1,000 (L)	17.0
*Pinus roxburghii*	1,500 (H)	81.0
*Pinus wallichiana*	2,550 (L)	50.0
*Pinus gerardiana*	2,850 (H)	349.4
*Quercus floribunda*	2,150 (L)	3,120.0
*Quercus semecarpifolia*	2,850 (H)	7,143.0
*Quercus glauca*	1,300 (L)	1,492.0
*Quercus lamellosa*	2,500 (H)	9,523.8
*Rhododendron arboreum*	2,000 (L)	0.02
*Rhododendron hodgsonii*	3,350 (H)	0.24

### 3.4 Seed mass variation in relation to habitat moisture, succession, and desiccation behavior

**Figure 5:**
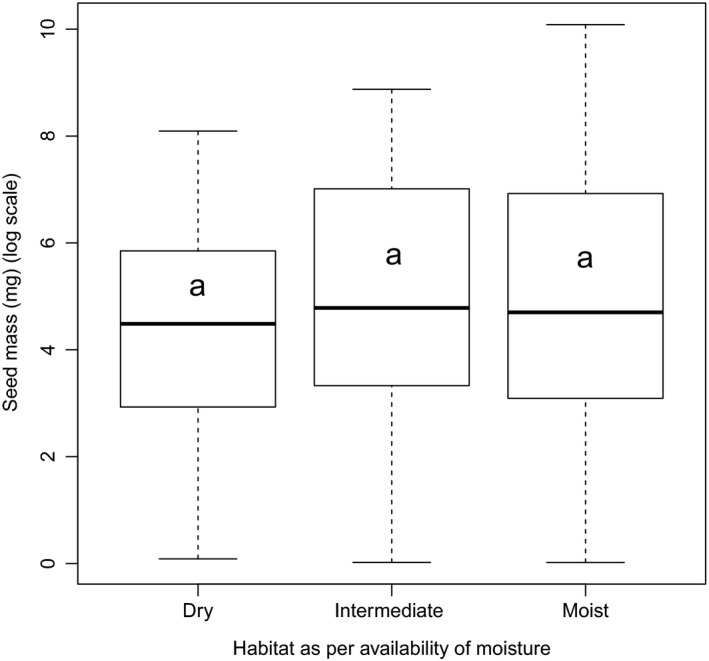
Box plots illustrating median and quartiles of mean seed mass of tree species occurring in moist (*n* = 85), intermediate (*n* = 17), and dry (*n* = 64) habitats. The same letter represents subsets with nonsignificant (*p* *>* .05) differences

**Figure 6:**
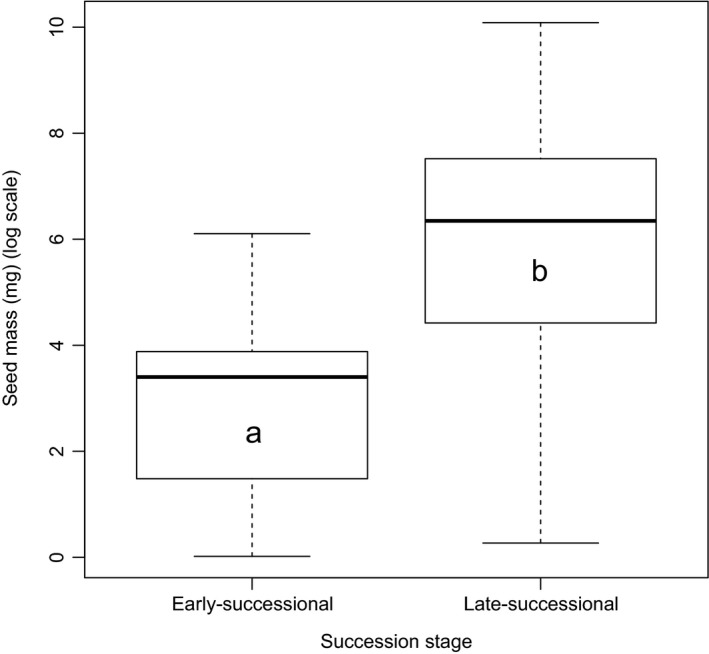
Box plots illustrating median and quartiles of mean seed mass in tree species of early‐ (*n* = 35) and late‐successional (*n* = 76) status. Letters represent subsets with significant (*p* < .001) differences

**Figure 7:**
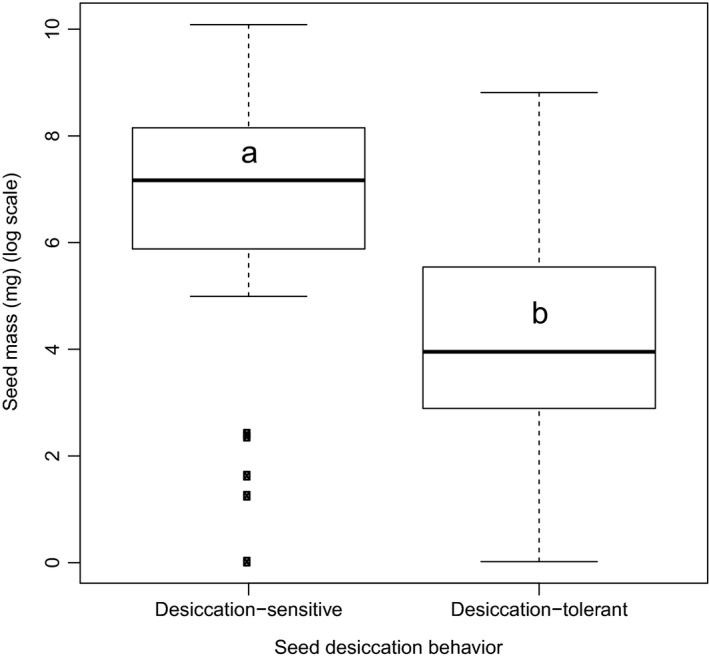
Box plots illustrating median, quartiles, and outliers (o) of mean seed mass of desiccation‐sensitive (*n* = 41) and desiccation‐tolerant (*n* = 149) species. Letters represent subsets with significant (*p* < .001) differences

Average seed mass in species of moist habitats (1,491 ± 449 mg/seed; *n* = 85) was about 1.8 times greater than in intermediate habitat (836 ± 740 mg/seed; *n* = 17) and 3.8 times greater than that of dry habitats (395 ± 339 mg/seed; *n* = 64). However, the results of the GLS regression revealed that seed mass of tree species occurring in all habitats was statistically at par (Fig. 5). The difference in average seed mass between late‐successional tree species (1,774 ± 625 mg/seed; *n* = 76) and early‐successional tree species (56 ± 517 mg/seed; *n* = 35) was found to be significant (Fig. 6), and occurrence of desiccation‐sensitive seeds was highly skewed (89%) in favor of the late‐successional tree species. We found that mean seed mass of tree species with desiccation‐sensitive seeds (3,095 ± 370 mg/seed; *n* = 41) were significantly larger (Fig. 7) than that of desiccation‐tolerant seeds (388 ± 418 mg/seed; *n* = 149). Of the 158 tree species for which information about both habitat moisture and desiccation behavior were available, overall about 52% and 38% were distributed in moist and dry habitats, respectively. However, of 33 desiccation‐sensitive species, majority (79%) occurred in moist habitats (Table 5).

## 4 DISCUSSION

Our study is the first attempt to shed light on the response of tree seed traits to the monsoonal pattern of precipitation, altitudinal gradient, habitat moisture, and tree successional stage to reveal how desiccation‐sensitive seed‐bearing tree species have the ecological domination in terms of geographical area of coverage. More specifically, we found out that the tree species of Himalayas and adjoining plains follow three synchronization strategies of seed shedding and seed germination timing with monsoon: MS (in 23% species), PMS (in 57% species), and MD (in 20% species). Synchronization of seed germination with the monsoon is a principal adaptation strategy that 80% of species (MS and PMS) employ to maximize seedling establishment. In the dryland environment and seasonal climate generally, seeds are shed at the time of maximum annual precipitation (Pritchard et al., 2004). A similar synchronization strategy with monsoon has also been found in 26 of 36 tree species of seasonally dry tropical forests of northern Thailand (Blakesley et al., 2002). The proportion of species (80% of MS and PMS) of the present study in which seed germination occurs in monsoon season is almost similar to that reported {82% in RRS (rapid‐rainy syndrome) and IDS (intermediate‐dry syndrome)} by Garwood (1983) for seed‐based regeneration syndromes in the study of Barro Colorado Island, Panama. Seasonality is believed to be more evident in a tropical dry forest, where the dry season has a strong impact on synchronization of seed dispersal and seed germination to limit their seedling emergence in the rainy season (Garwood, 1983; Vieira & Scariot, 2006), as also verified in the present results. It is expected that seasonality might have a weaker impact on seed rain and seedling emergence in a rain forest. For example, seedling emergence has been observed throughout the year in wet forests of Malaysia (Ng, 1978) and Nigeria (Okali & Onyeachusim, 1991). In evergreen rain forests, species with desiccation‐sensitive seeds represent about 47% of woody plant species (Tweddle et al., 2003), which is almost similar (45%; 18 of 40 species) to that of the desiccation‐sensitive seeds of MS species of the present study (Table 1). This confirms our first hypothesis to some extent that the percentage of species with desiccation‐sensitive seeds will be higher in the species group in which monsoon linkage is strong as 92% of desiccation‐sensitive seed‐bearing study species synchronized with monsoon either fully (45% of MS) or partially (47% of PMS). The climatic condition during four monsoon months in many parts of the Himalayas and adjoining plains is like that of rain forests. Of the overall tree species of the present study for which information about seed desiccation behavior is available, about 21% (40 of 188 species) have desiccation‐sensitive seeds which is similar to that of woody species of temperate moist forests (23.3%) and tropical semievergreen rain forest (20.6%) (Tweddle et al., 2003).

Between the two principal categories of monsoon synchronization, seed mass was 2.7 times greater for MS than PMS species (Fig. 2). This is consistent with the finding of Murali (1997), based on 99 monsoonal tropical tree species of southern India, that species which fruit during a dry period have lighter seeds mass than those which fruit during a wet period. The large difference in mean seed mass of desiccation‐sensitive (3,958 mg/seed) and desiccation‐tolerant species (329 mg/seed) was reported by Dickie and Pritchard (2002) for global species data pool and later verified by Daws et al. (2005) and Daws, Cleland, et al. (2006). Results of our study are in line with their findings as seed mass of desiccation‐sensitive species in our sample was about eight times more than that of desiccation‐tolerant seed‐bearing species (Fig. 7), and the majority (79%) of these desiccation‐sensitive species were distributed in moist habitat (Table 6). This confirms our second hypothesis that the average seed mass will be greater in desiccation‐sensitive species, hence in those species in which monsoon synchronization is strong. The large seeds would lose seed moisture content relatively slowly, need to allocate less resources to seed coat and/or endocarp (Daws et al., 2005), and may confer better survival in deep shade (Forget, 1992; Foster, 1986; Harms & Dalling, 1997; Leishman & Westoby, 1994a,b; Pritchard et al., 2004; Westoby, Leishman, & Lord, 1996).

**Table 6:** Species number by seed desiccation behavior and habitat moisture types

Habitat moisture type	Desiccation‐sensitive seeds (no. and %)	Desiccation‐tolerant seeds (no. and %)	Total (no. and %)
Moist	26 (78.9)	57 (45.6)	83 (52.5)
Intermediate	04 (12.1)	11 (8.8)	15 (9.5)
Dry	03 (9.0)	57 (45.6)	60 (38.0)
Grand total	33 (20.9)	125 (79.1)	158

In comparison with the MS species, desiccation‐sensitive seeds of the PMS species are exposed to longer periods of desiccation before initiation of germination in the monsoon in much of their distribution ranges. A majority of Himalayan oaks and species of *Castanopsis* and *Schima wallichii*, which dominate extensively between 1,000 and 2,800 m altitudes belong to the PMS category. These species avoid dry inner valleys and decrease in ecological dominance toward the west along the Himalayan Arc (Ohsawa, 1991; Singh & Singh, 1992). Nevertheless, seed desiccation limits seed germination in PMS species and it is getting intensified with global warming in some oaks (e.g., *Quercus leucotrichophora*), which are failing to regenerate in several locations in Central Himalayas for last few decades (Singh, 2014). The desiccation‐sensitive seeds of PMS species might restrict the loss of seed moisture by having protective pericarp components and anatomical arrangements. In a study on desiccation‐sensitive seeds of some oaks, the role of anatomical adaptations of the pericarp was shown in resisting water loss (Xia, Daws, Stuppy, Zhou, & Pritchard, 2012; Xia, Hill, Li, & Walters, 2014).

To cope with the long dry period between shedding and germination, 82.4% of PMS species of our study have desiccation‐tolerant seeds (Table 1) and the majority of them belong to *Fabaceae* known for having physically dormant seeds (Baskin & Baskin, 2014; Jayasuriya, Wijetunga, Baskin, & Baskin, 2013). Continual high or high fluctuating temperatures before the onset of monsoon trigger dormancy release in them. In some species of *Fabaceae*, seeds may germinate a couple of months after shedding, during monsoon months, but if they fail to germinate, then they remain on ground for another year or more, and germinate only during the following monsoon or next to that, for example, *Cassia fistula* and several species of *Bauhinia* (Troup, 1921). The advantage of seeds lying on ground over several months could be (1) an effective seed dispersal by animals (Champion & Seth, 1968; Troup, 1921), (2) the reduction in risk of losing whole of an annual cohort of seeds to drought or other adverse conditions because of temporal spreading of seed dispersal (Pritchard et al., 2004), and (3) the ensured availability of monsoon rain water at least to those seedlings which are recruited closer to the defined period of precipitation (Berjak & Pammenter, 2008; Farnsworth, 2000).

All the MD species, with the exception of *Aesculus indica*,* Quercus semiserrata*,* Q. serrata* and *Tsuga dumosa* are desiccation‐tolerant, and the majority of them belong to *Aceraceae*,* Betulaceae*,* Oleaceae*,* Pinaceae*, and *Taxaceae* that are known for physiologically or morphophysiologically dormant seeds (Baskin & Baskin, 2014). Almost all MD species shed seeds in autumn are cold stratified during winter and eventually germinate by early summer when both moisture and temperatures conditions become conducive for seedlings survival. In a way, species of this group employ desiccation tolerance and dormancy of seeds to achieve a monsoon‐desynchronized strategy. While desiccation‐sensitive seed‐bearing species in this category might have some protective pericarp components to restrict the loss of seed moisture like in seeds of some oaks (Xia et al., 2012, 2014) in addition to dormancy.

It is argued that seed mass is an important trait which provides a competitive advantage to species under stressful drought conditions (Daws et al., 2007; Leishman & Westoby, 1994b; Westoby et al., 1996). However, when seed germination and seedling establishment of eight dipterocarps were subjected to variable rainfall frequency in Borneo, Malaysia, large‐seeded species showed an establishment advantage when water was nonlimiting, but the growth of large‐seeded species was more inhibited under infrequent rainfall than the growth of small‐seeded species. This indicates the timing of rainless period (before or after germination) might alter the competitive advantage of large‐seeded species (O'Brien, Philipson, Tay, & Hector, 2013). It may also be applicable to the tree species of the Himalayas and adjoining plains that whenever a delay in monsoon or a prolonged break‐spell of rain during monsoon occurs, seed‐based regeneration in large‐seeded MS species will be affected more adversely than in small‐seeded PMS species.

The correlation between seed mass and altitude is reported to be both positive (Pluess et al., 2005; Vera, 1997) and negative (Bu et al., 2007; Counts & Lee, 1991). We found that the overall seed mass was not correlated with altitude. In contrast, within the pairs of related species differing in altitude in the Himalayas, the species of higher altitudes generally had relatively greater seed mass (Table 5). Species with large seeds may be selected in stressful conditions of high‐altitude areas because they have large food reserves in seeds to establish seedlings. Pluess et al. (2005) found similar pattern while comparing 29 cogeneric species pairs from low‐ and high‐altitude areas of Swiss Alps. While analyzing the correlation between seed mass and altitude, we have also separated species on the basis of the sensitivity of seeds to desiccation and monsoon synchronization. We found no relationship between seed mass and altitude on the basis of desiccation behavior (Fig. 3). However, seed mass varied significantly along altitude as per monsoon synchronization strategy and correlated positively for MS and PMS and negatively for MD (Fig. 4). Although some of the species with desiccation‐sensitive seeds (e.g., *Aesculus* and *Quercus* spp.) occurred above 2,000 m, their upper altitudinal limit (3,000 m) was distinctly lower than that of desiccation‐tolerant seed‐bearing species. To some degree, this is in line with our third hypothesis that the upper altitudinal limit of desiccation‐tolerant seed‐bearing species will be high.

We found no phylogenic signals in any of species groups with seed mass, indicating ecology, rather than phylogeny of study tree species is better correlated with species monsoon synchronization strategies. As the outer Himalayan ranges are exposed to the direct thrust of monsoon, growth conditions in them remain favorable up to 3,000 m. For example, nutrient turnover rates in forests of those altitudes are closer to those of tropical forests than of forests of temperate regions (Zobel & Singh, 1997). There is an association between late‐successional moist tropical forest trees with large, moist, nondormant, and desiccation‐sensitive seeds (Dickie & Pritchard, 2002; Farnsworth, 2000; Vázquez‐Yanes & Orozco‐Segovia, 1984; Vázquez‐Yanes et al., 2000), while species from dry arid or highly seasonal habitats are overwhelmingly dominated by species producing desiccation‐tolerant seeds (Hong, Linington, & Ellis, 1998; Roberts & King, 1980). In the present study, we found higher mean seed mass and high frequency of desiccation‐sensitive seeds in late‐successional species than in early‐successional species, which proves our final hypothesis. The similar trend has also been observed for other biogeographical regions (Daws et al., 2002; Garwood, 1989; Grime, Hodgson, & Hunt, 1988; Moles et al., 2005, 2007; Salisbury, 1974; Tweddle et al., 2002). It is also observed that average seed mass in species of moist habitats was about 3.8 times greater than that of dry habitats; however, this difference found statistically at par that indicates seed mass is not correlated with habitat moisture. Almost similar trend reported for Californian trees or shrubs species by Baker (1972), for 648 species of the Indiana dunes by Mazer (1990), and for 14 oak species by Long and Jones (1996).

## 5 CONCLUSIONS

This study focused on identifying seed shedding and germination trends with respect to seasonality (synchrony with monsoons), altitude, and habitat to provide some new insights into the relationship between seed desiccation behavior and timing of seed germination. Majority of the study tree species irrespective to seed desiccation behavior show an adaptation of seed germination synchronization with monsoon rainfall. However, some of the species with desiccation‐sensitive seeds nonetheless still persist without synchronizing seed shedding and germination with the monsoon, and that warrants further in‐depth investigations how they cope desiccation damage during a dry spell. We also need to know how desiccation‐sensitive seeds of MS and PMS species react if a delay in monsoon or a prolonged break‐spell of monsoon rain occurs. As increased evapotranspiration loss and the depletion of snowmelt water due to the rise in temperature at higher rates than global average rate in Himalayan regions (Shrestha et al., 2012), coupled with weakened monsoon (Bhuiyan et al., 2009; Yao et al., 2012), seem to have intensified both pre‐ and post‐monsoon droughts (Liang, Dawadi, Pederson, & Eckstein, 2014), this scenario might adversely affect the early life cycle events in the majority of species studied. Thus, a well‐informed knowledge of seed‐based regeneration ecology of Himalayan tree species will improve our understanding of the desiccation‐sensitive seed‐bearing species dominance over large forest areas of monsoonal climate.

## CONFLICT OF INTEREST

None declared.
