# Supplementary figures and images for: Tree seed traits’ response to monsoon climate and altitude in Indian subcontinent with particular reference to the Himalayas
Source: Ecol Evol. 2017 Aug 11;7(18):7408–19. doi: 10.1002/ece3.3181 (PMC5606906; doi:10.1002/ece3.3181)

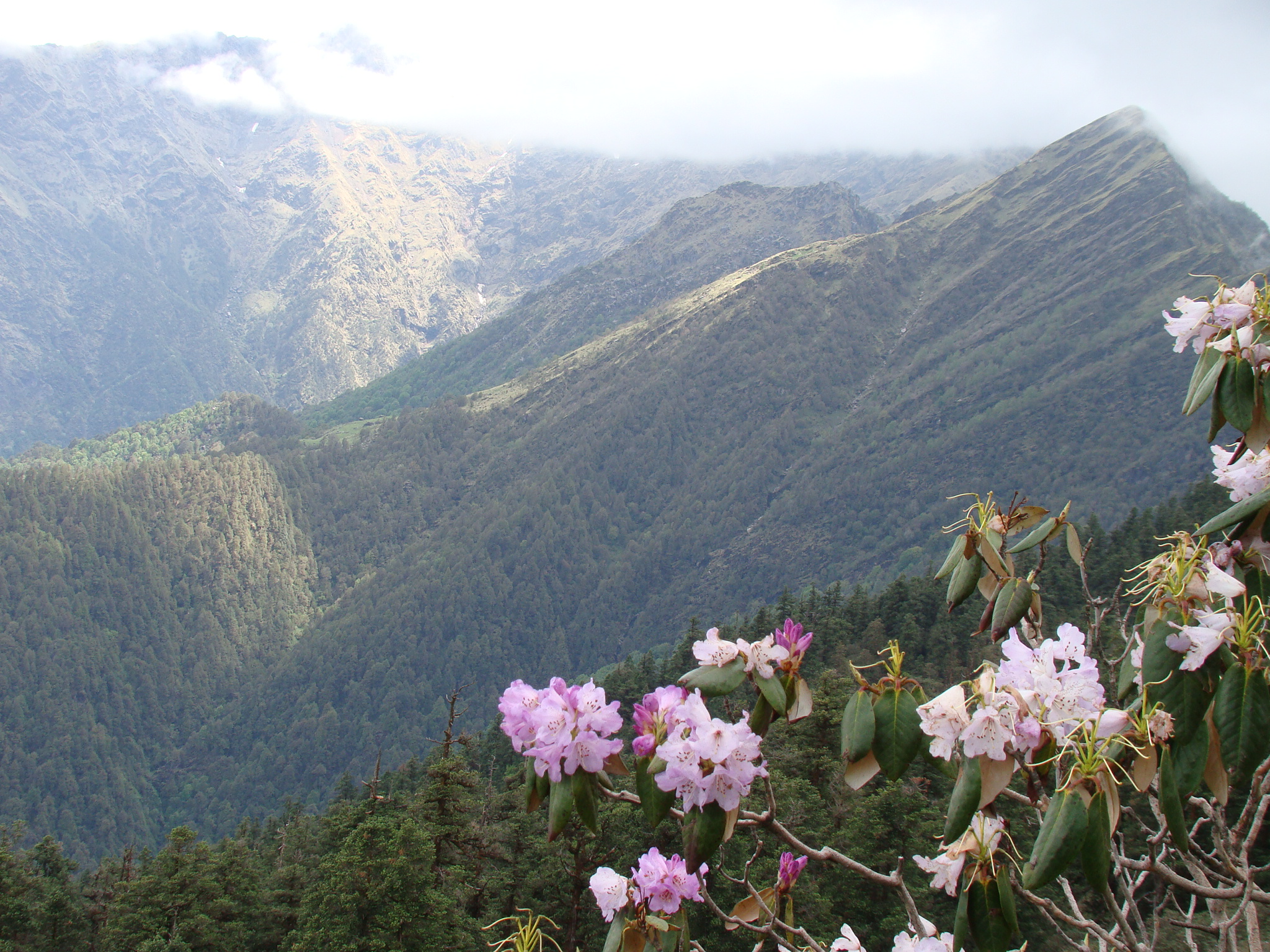

Supplement: Supplementary file 1 [file ECE3-7-7408-s001.JPG]
