# Supplementary material for: Tree seed traits’ response to monsoon climate and altitude in Indian subcontinent with particular reference to the Himalayas
Source: Ecol Evol. 2017 Aug 11;7(18):7408–19. doi: 10.1002/ece3.3181 (PMC5606906; doi:10.1002/ece3.3181)
Supplement: Supplementary file 2 [file ECE3-7-7408-s002.docx]

**Supplementary Appendix 1:** List of study species with scientific name (including author) and family.

| **S. No.** | **Species** | **Family** |
| --- | --- | --- |
| 1 | *Abies pindrow* (Royle ex D.Don) Royle | Pinaceae |
| 2 | *Abies spectabilis* (D.Don) Mirb. | Pinaceae |
| 3 | *Acacia catechu* (L. f.) Willd. | Fabaceae |
| 4 | *Acacia modesta* (Wall.) P. Hurter | Fabaceae |
| 5 | *Acacia nilotica* (L.) Willd. ex Delile | Fabaceae |
| 6 | *Acer caesium* Wall. ex Brandis | Sapindaceae |
| 7 | *Acer campbellii* Hook.f. & Thomson ex Hiern | Sapindaceae |
| 8 | *Acer cappadocicum* Gled. | Sapindaceae |
| 9 | *Acer oblongum* Wall. ex DC. | Sapindaceae |
| 10 | *Acer pictum* Thunb. | Sapindaceae |
| 11 | *Acer stachyophyllum* Hiern | Sapindaceae |
| 12 | *Acer sterculiaceum* Wall. | Sapindaceae |
| 13 | *Acrocarpus fraxinifolius* Arn. | Fabaceae |
| 14 | *Adina cordifolia* (Roxb.) Ridsdale | Rubiaceae |
| 15 | *Aegle marmelos* (L.) Corrêa | Rutaceae |
| 16 | *Aesculus indica* (Wall. ex Cambess.) Hook. | Sapindaceae |
| 17 | *Aglaia spectabilis* (Miq.) S.S.Jain & S.Bennet | Meliaceae |
| 18 | *Ailanthus excelsa* Roxb. | Simaroubaceae |
| 19 | *Albizia chinensis* (Osbeck)Merr. | Fabaceae |
| 20 | *Albizia lebbeck* (L.)Benth. | Fabaceae |
| 21 | *Albizia odoratissima* (L.f.)Benth. | Fabaceae |
| 22 | *Albizia procera* (Roxb.)Benth. | Fabaceae |
| 23 | *Alnus nepalensis* D.Don | Betulaceae |
| 24 | *Alnus nitida (*Spach) Endl. | Betulaceae |
| 25 | *Anogeissus acuminata* (Roxb. ex DC.) Wall. ex Guillem. & Perr. | Combretaceae |
| 26 | *Anogeissus latifolia* (Roxb. ex DC.) Wall. ex Guillem. & Perr. | Combretaceae |
| 27 | *Anogeissus pendula* Edgew. | Combretaceae |
| 28 | *Aphanamixis polystachya* (Wall.) R.Parker | Meliaceae |
| 29 | *Artocarpus chama* Buch.-Ham. | Moraceae |
| 30 | *Artocarpus lakoocha* Buch.-Ham. | Moraceae |
| 31 | *Azadirachta indica* A.Juss. | Meliaceae |
| 32 | *Bassia eriophora* (Schrad.) Asch. | Amaranthaceae |
| 33 | *Bauhinia purpurea* L*.* | Fabaceae |
| 34 | *Bauhinia racemosa* Lam. | Fabaceae |
| 35 | *Bauhinia retusa* Roxb. | Fabaceae |
| 36 | *Bauhinia variegata* L. | Fabaceae |
| 37 | *Betula alnoides* Buch.-Ham. ex D.Don | Betulaceae |
| 38 | *Betula utilis* D.Don | Betulaceae |
| 39 | *Bischofia javanica* Blume | Phyllanthaceae |
| 40 | *Bombax ceiba* L. | Malvaceae |
| 41 | *Boswellia serrata* Roxb. ex Colebr. | Burseraceae |
| 42 | *Buchanania cochinchinensis* (Lour.) M.R.Almeida | Anacardiaceae |
| 43 | *Butea monosperma* (Lam.)Taub. | Fabaceae |
| 44 | *Buxus wallichiana* Baill. | Buxaceae |
| 45 | *Calophyllum polyanthum* Wall. ex Planch. & Triana | Calophyllaceae |
| 46 | *Careya arborea* Roxb. | Lecythidaceae |
| 47 | *Casearia graveolens* Dalzell | Salicaceae |
| 48 | *Cassia fistula* L. | Fabaceae |
| 49 | *Castanea dentata* (Marshall) Borkh. | Fagaceae |
| 50 | *Castanopsis indica* (Roxb. ex Lindl.) A.DC. | Fagaceae |
| 51 | *Castanopsis tribuloides* (Sm.) A.DC. | Fagaceae |
| 52 | *Cedrus deodara* (Roxb. ex D.Don) G.Don | Pinaceae |
| 53 | *Celtis australis* L. | Cannabaceae |
| 54 | *Chukrasia tabularis* A.Juss. | Meliaceae |
| 55 | *Cochlospermum religiosum* (L.) Alston | Bixaceae |
| 56 | *Cordia myxa* L. | Boraginaceae |
| 57 | *Cordia vestita* (A.DC.) Hook.f. & Thomson | Boraginaceae |
| 58 | *Cornus macrophylla* Wall. | Cornaceae |
| 59 | *Cotoneaster frigidus* Wall. ex Lindl. | Rosaceae |
| 60 | *Crateva religiosa* G.Forst. | Capparaceae |
| 61 | *Cupressus torulosa* D.Don | Cupressaceae |
| 62 | *Dalbergia latifolia* Roxb. | Fabaceae |
| 63 | *Dalbergia sissoo* DC. | Fabaceae |
| 64 | *Desmodium oojeinense* (Roxb.)H.Ohashi | Fabaceae |
| 65 | *Dillenia indica* L. | Dilleniaceae |
| 66 | *Dillenia pentagyna* Roxb. | Dilleniaceae |
| 67 | *Diospyros malabarica* (Desr.) Kostel. | Ebenaceae |
| 68 | *Diospyros melanoxylon* Roxb. | Ebenaceae |
| 69 | *Dipterocarpus gracilis* Blume | Dipterocarpaceae |
| 70 | *Dipterocarpus retusus* Blume | Dipterocarpaceae |
| 71 | *Dipterocarpus tuberculatus* Roxb. | Dipterocarpaceae |
| 72 | *Dipterocarpus turbinatus* C.F.Gaertn | Dipterocarpaceae |
| 73 | *Duabanga grandiflora* (DC.) Walp. | Lythraceae |
| 74 | *Ehretia laevis* Roxb. | Boraginaceae |
| 75 | *Erythrina suberosa* Roxb. | Fabaceae |
| 76 | *Feronia limonia* (L.) Swingle | Rutaceae |
| 77 | *Ficus benghalensis* L. | Moraceae |
| 78 | *Ficus elastica* Roxb. ex Hornem. | Moraceae |
| 79 | *Ficus hirta subsp. roxburghii (King) C.C.Berg* | Moraceae |
| 80 | *Ficus minahassae* (Teijsm. & Vriese) Miq. | Moraceae |
| 81 | *Ficus palmata* Forssk. | Moraceae |
| 82 | *Ficus religiosa* L. | Moraceae |
| 83 | *Ficus retusa* L. | Moraceae |
| 84 | *Ficus semicordata* Buch.-Ham. ex Sm. | Moraceae |
| 85 | *Ficus tinctoria subsp. gibbosa* (Blume) Corner | Moraceae |
| 86 | *Firmiana colorata* (Roxb.) R.Br. | Malvaceae |
| 87 | *Fraxinus excelsior* L. | Oleaceae |
| 88 | *Fraxinus micrantha* Lingelsh. | Oleaceae |
| 89 | *Fraxinus xanthoxyloides* (G.Don) Wall. ex A.DC. | Oleaceae |
| 90 | *Garuga pinnata* Roxb. | Burseraceae |
| 91 | *Gmelina arborea* Roxb. | Lamiaceae |
| 92 | *Grewia elastica* Juss. | Malvaceae |
| 93 | *Grewia optiva* J.R.Drumm. ex Burret | Malvaceae |
| 94 | *Grewia tiliifolia* Vahl | Malvaceae |
| 95 | *Hardwickia binata* Roxb. | Fabaceae |
| 96 | *Helicteres isora* L. | Malvaceae |
| 97 | *Holarrhena antidysenterica* Wall. ex G.Don | Apocynaceae |
| 98 | *Hydnocarpus kurzii* (King) Warb. | Achariaceae |
| 99 | *Hymenodictyon orixense* (Roxb.) Mabb. | Rubiaceae |
| 100 | *Ilex dipyrena* Wall. | Aquifoliaceae |
| 101 | *Jasminum humile* L. | Oleaceae |
| 102 | *Juglans regia* L. | Juglandaceae |
| 103 | *Juniperus communis* L. | Cupressaceae |
| 104 | *Juniperus polycarpos var. seravschanica* (Kom.) Kitam. | Cupressaceae |
| 105 | *Juniperus pseudosabina* Fisch. & C.A.Mey. | Cupressaceae |
| 106 | *Juniperus recurva* Buch.-Ham. ex D.Don | Cupressaceae |
| 107 | *Lagerstroemia parviflora* Roxb. | Lythraceae |
| 108 | *Lagerstroemia speciosa* (L.) Pers. | Lythraceae |
| 109 | *Lagerstroemia tomentosa* C. Presl | Lythraceae |
| 110 | *Lannea coromandelica* (Houtt.) Merr. | Anacardiaceae |
| 111 | *Litsea glutinosa* (Lour.) C.B.Rob. | Lauraceae |
| 112 | *Litsea monopetala* (Roxb.) Pers. | Lauraceae |
| 113 | *Litsea umbrosa* (Nees) Gamble | Lauraceae |
| 114 | *Lyonia ovalifolia* (Wall.) Drude | Ericaceae |
| 115 | *Magnolia campbellii* Hook.f. & Thomson | Magnoliaceae |
| 116 | *Mallotus philippensis* (Lam.) Müll.Arg. | Euphorbiaceae |
| 117 | *Malus baccata* (L.) Borkh. | Rosaceae |
| 118 | *Mangifera indica* L. | Anacardiaceae |
| 119 | *Melicope lunu-ankenda* (Gaertn.) T.G. Hartley | Rutaceae |
| 120 | *Mesua ferrea* L. | Calophyllaceae |
| 121 | *Michelia champaca* (L.) Baill. ex Pierre | Magnoliaceae |
| 122 | *Miliusa longiflora* Hook.f. & Thomson | Annonaceae |
| 123 | *Miliusa velutina* (A.DC.) Hook.f. & Thomson | Annonaceae |
| 124 | *Murraya koenigii*(L.) Spreng. | Rutaceae |
| 125 | *Neolamarckia cadamba* (Roxb.) Bosser | Rubiaceae |
| 126 | *Olea europaea subsp. cuspidata* (Wall. & G.Don) Cif. | Oleaceae |
| 127 | *Oroxylum indicum* (L.) Kurz | Bignoniaceae |
| 128 | *Persea odoratissima (*Nees) Kosterm. | Lauraceae |
| 129 | *Phoebe lanceolata* (Schott) Mez | Lauraceae |
| 130 | *Phyllanthus emblica* L. | Phyllanthaceae |
| 131 | *Picea smithiana* (Wall.) Boiss. | Pinaceae |
| 132 | *Picea spinulosa* (Griff.) A.Henry | Pinaceae |
| 133 | *Pinus gerardiana* Wall. ex D.Don | Pinaceae |
| 134 | *Pinus kesiya* Royle ex Gordon | Pinaceae |
| 135 | *Pinus merkusii* Jungh. & de Vriese | Pinaceae |
| 136 | *Pinus roxburghii* Sarg. | Pinaceae |
| 137 | *Pinus wallichiana* A.B.Jacks. | Pinaceae |
| 138 | *Podocarpus neriifolius* D.Don | Podocarpaceae |
| 139 | *Polyalthia simiarum* (Buch.-Ham. ex Hook. f. & Thomson) Benth. ex Hook. f. & Thomson | Annonaceae |
| 140 | *Populus ciliata* Wall. ex Royle | Salicaceae |
| 141 | *Prunus cerasoides* Buch.-Ham. ex D.Don | Rosaceae |
| 142 | *Prunus padus* L. | Rosaceae |
| 143 | *Pterocarpus macrocarpus* Kurz | Fabaceae |
| 144 | *Pterocarpus marsupium* Roxb. | Fabaceae |
| 145 | *Pterospermum acerifolium* (L.) Willd. | Malvaceae |
| 146 | *Pterygota alata* (Roxb.) R.Br. | Malvaceae |
| 147 | *Pyrus pashia* Buch.-Ham. ex D.Don | Rosaceae |
| 148 | *Quercus floribunda* Lindl. ex A.Camus | Fagaceae |
| 149 | *Quercus glauca* Thunb. | Fagaceae |
| 150 | *Quercus griffithii* Hook.f. & Thomson ex Miq. | Fagaceae |
| 151 | *Quercus ilex* L. | Fagaceae |
| 152 | *Quercus lamellosa* Sm*.* | Fagaceae |
| 153 | *Quercus lanata* Sm. | Fagaceae |
| 154 | *Quercus leucotrichophora* D.Don | Fagaceae |
| 155 | *Quercus lineata* Blume | Fagaceae |
| 156 | *Quercus oblongata* D.Don | Fagaceae |
| 157 | *Quercus semecarpifolia* Sm. | Fagaceae |
| 158 | *Quercus semiserrata* Roxb. | Fagaceae |
| 159 | *Quercus serrata* Murray | Fagaceae |
| 160 | *Rhododendron arboreum* Sm. | Ericaceae |
| 161 | *Rhododendron hodgsonii* Hook. f. | Ericaceae |
| 162 | *Saccopetalum longiflorum* Hook.f. & Thomson | Magnoliaceae |
| 163 | *Sapindus emarginatus* Vahl | Sapindaceae |
| 164 | *Sapindus mukorossi* Gaertn. | Sapindaceae |
| 165 | *Schima wallichii* Choisy | Theaceae |
| 166 | *Schleichera oleosa* (Lour.) Merr. | Sapindaceae |
| 167 | *Shorea assamica* Dyer | Dipterocarpaceae |
| 168 | *Shorea robusta* Gaertn. | Dipterocarpaceae |
| 169 | *Sorbus cuspidata* (Spach) Hedl. | Rosaceae |
| 170 | *Sorbus foliolosa* (Wall.) Spach | Rosaceae |
| 171 | *Sorbus microphylla* (Wall. ex Hook.f.) Wenz*.* | Rosaceae |
| 172 | *Spondias pinnata* (L. f.) Kurz | Anacardiaceae |
| 173 | *Sterculia foetida* L*.* | Malvaceae |
| 174 | *Sterculia pallens* Wall. ex Hochr*.* | Malvaceae |
| 175 | *Sterculia urens* (L.) W.Wight | Malvaceae |
| 176 | *Sterculia villosa* Roxb. | Malvaceae |
| 177 | *Stereospermum chelonoides* (L.f.) DC. | Bignoniaceae |
| 178 | *Strychnos nux-vomica* L. | Loganiaceae |
| 179 | *Symplocos paniculata* Miq. | Symplocaceae |
| 180 | *Syzygium cumini* (L.) Skeels | Myrtaceae |
| 181 | *Tamarindus indica* L. | Fabaceae |
| 182 | *Tamarix aphylla* (L.) H.Karst. | Tamaricaceae |
| 183 | *Taxus baccata* L. | Taxaceae |
| 184 | *Tectona grandis* L.f. | Lamiaceae |
| 185 | *Terminalia alata* D.Dietr. | Combretaceae |
| 186 | *Terminalia arjuna* (Roxb. ex DC.) Wight & Arn. | Combretaceae |
| 187 | *Terminalia bellirica* (Gaertn.) Roxb. | Combretaceae |
| 188 | *Terminalia chebula* Retz. | Combretaceae |
| 189 | *Terminalia myriocarpa* Van Heurck & Müll. Arg*.* | Combretaceae |
| 190 | *Terminalia tomentosa* Wight & Arn. | Combretaceae |
| 191 | *Toona ciliata* M.Roem. | Meliaceae |
| 192 | *Toona sinensis* (Juss.) M.Roem. | Meliaceae |
| 193 | *Toona sureni* (Blume) Merr. | Meliaceae |
| 194 | *Trewia nudiflora* L. | Euphorbiaceae |
| 195 | *Tsuga dumosa* (D.Don) Eichler | Pinaceae |
| 196 | *Ulmus integrifolia* Roxb*.* | Ulmaceae |
| 197 | *Ulmus wallichiana* Planch. | Ulmaceae |
| 198 | *Wrightia arborea* (Dennst.) Mabb. | Apocynaceae |
